# Introducing AI to the molecular tumor board: one direction toward the establishment of precision medicine using large-scale cancer clinical and biological information

**DOI:** 10.1186/s40164-022-00333-7

**Published:** 2022-10-31

**Authors:** Ryuji Hamamoto, Takafumi Koyama, Nobuji Kouno, Tomohiro Yasuda, Shuntaro Yui, Kazuki Sudo, Makoto Hirata, Kuniko Sunami, Takashi Kubo, Ken Takasawa, Satoshi Takahashi, Hidenori Machino, Kazuma Kobayashi, Ken Asada, Masaaki Komatsu, Syuzo Kaneko, Yasushi Yatabe, Noboru Yamamoto

**Affiliations:** 1 Division of Medical AI Research and Development, National Cancer Center Research Institute, 5-1-1 Tsukiji, Chuo-ku, Tokyo, 104-0045 Japan; 2 Cancer Translational Research Team, RIKEN Center for Advanced Intelligence Project, 1-4-1 Nihonbashi, Chuo-ku, Tokyo, 103-0027 Japan; 3 Department of Experimental Therapeutics, National Cancer Center Hospital, 5-1-1 Tsukiji, Chuo-ku, Tokyo, 104-0045 Japan; 4 Department of Surgery, Graduate School of Medicine, Kyoto University, Yoshida-konoe-cho, Sakyo-ku, Kyoto, 606-8303 Japan; 5 Research and Development Group, Hitachi, Ltd., 1-280 Higashi-koigakubo, Kokubunji, Tokyo, 185-8601 Japan; 6 Department of Medical Oncology, National Cancer Center Hospital, 5-1-1 Tsukiji, Chuo-ku, Tokyo, 104-0045 Japan; 7 Department of Genetic Medicine and Services, National Cancer Center Hospital, 5-1-1 Tsukiji, Chuo-ku, Tokyo, 104-0045 Japan; 8 Department of Laboratory Medicine, National Cancer Center Hospital, 5-1-1 Tsukiji, Chuo-ku, Tokyo, 104-0045 Japan; 9 Department of Diagnostic Pathology, National Cancer Center Hospital, 5-1-1 Tsukiji, Chuo-ku, Tokyo, 104-0045 Japan; 10 Division of Molecular Pathology, National Cancer Center Research Institute, 5-1-1 Tsukiji, Chuo-ku, Tokyo, 104-0045 Japan

**Keywords:** Molecular tumor board, Precision medicine, Artificial intelligence, Next-generation sequencing, Natural language processing

## Abstract

Since U.S. President Barack Obama announced the Precision Medicine Initiative in his New Year’s State of the Union address in 2015, the establishment of a precision medicine system has been emphasized worldwide, particularly in the field of oncology. With the advent of next-generation sequencers specifically, genome analysis technology has made remarkable progress, and there are active efforts to apply genome information to diagnosis and treatment. Generally, in the process of feeding back the results of next-generation sequencing analysis to patients, a molecular tumor board (MTB), consisting of experts in clinical oncology, genetic medicine, etc., is established to discuss the results. On the other hand, an MTB currently involves a large amount of work, with humans searching through vast databases and literature, selecting the best drug candidates, and manually confirming the status of available clinical trials. In addition, as personalized medicine advances, the burden on MTB members is expected to increase in the future. Under these circumstances, introducing cutting-edge artificial intelligence (AI) technology and information and communication technology to MTBs while reducing the burden on MTB members and building a platform that enables more accurate and personalized medical care would be of great benefit to patients. In this review, we introduced the latest status of elemental technologies that have potential for AI utilization in MTB, and discussed issues that may arise in the future as we progress with AI implementation.

## Background

The human genome project, which began in 1990 to analyze the entire human sequence, was declared complete in April 2003 after 13 years and a budget of approximately US$3 billion [1–3]. The world then entered the post-genomic era, and expectations grew for the development of “personalized medicine,“ in which genomic information is applied to medical treatment [4–6]. When the 454 Genome Sequencer 20 (GS20), the first next-generation sequencing (NGS) technology, was introduced in 2005, genetic analysis using NGS became actively pursued. Further, research in fields such as genetic medicine and pharmacogenomics became more active toward the realization of personalized medicine, which aims to provide medical care based on an individual’s genetic information [7–9]. In 2014, Illumina made genetic analysis increasingly accessible with the announcement of a $1,000 genome offer upon the sale of the HiSeq X Ten [10, 11]. Under these circumstances, the precision medicine initiative was announced by U.S. President Barack Obama in his New Year’s State of the Union address on January 20, 2015. The presentation declared that the study of how genomic information, environmental factors, and lifestyle affect health maintenance and disease development using large clinical samples will divide patients/potential patients into subgroups with respect to disease susceptibility and develop appropriate treatment and disease prevention methods for each group [12–14]. President Obama’s announcement impacted global healthcare policy, and the establishment of precision medicine systems was prioritized in countries globally. In particular, the U.S. FDA’s approval of MSK-IMPACT™ and FoundationOne® CDx, tumor profiling tests for solid tumors based on NGS-based genetic mutation analysis, in late 2017 increased momentum for optimal treatment based on genetic information in actual clinical practice [15–17]. In Japan, the OncoGuide™ NCC Oncopanel system and FoundationOne® CDx were approved by the Ministry of Health, Labour and Welfare in 2018 and covered by insurance in 2019, making cancer genomic medicine available under insurance reimbursement [18–20]. On the other hand, the molecular tumor board (MTB), which is composed of experts in various fields such as clinical oncology and genetic medicine, discusses the results of genetic mutation analysis; however, it is a complex, time-consuming, and labor-intensive process [21–23]. With the growing expectations for precision medicine and the need to make MTBs more efficient and effective, several MTBs have been reported that utilize a virtual environment [24–26]. The expectation and burden on MTB members are predicted to increase in the future, and the introduction of artificial intelligence (AI) and the latest information and communication technology (ICT) to improve the efficiency and automation of MTB is important when establishing a precision medicine system.

Along with recent advances in machine learning technology, particularly deep learning, AI technology has been attracting attention, and social implementation of AI is progressing in a variety of fields [27–29]. The medical field is no exception, with a succession of AI-based medical device programs being approved in countries globally, and their use in clinical settings is progressing [30–35]. Because deep learning technology is particularly strong in image analysis, AI research and development using medical images, such as radiological, endoscopic, ultrasound, and skin, has been actively conducted, and many important findings have been obtained [36–45]. In addition to medical image analysis, AI is used for omics data analysis and single cell analysis, and natural language processing (NLP), an AI technology, is now being used to analyze electronic medical records and medical papers, with research aimed at clinical applications [46–49].

This review focuses on the introduction of AI into the MTB and discusses the history of AI and the introduction of computers into diagnosis, the current status of AI utilization in the promotion of precision medicine, and future directions. Importantly, machine learning techniques, including NLP, are categorized as AI techniques because, as noted above, current AI techniques are based on machine learning methods.

## Machine learning, the technological foundation of current AI research and development, and its application to medical research

**Fig. 1:**
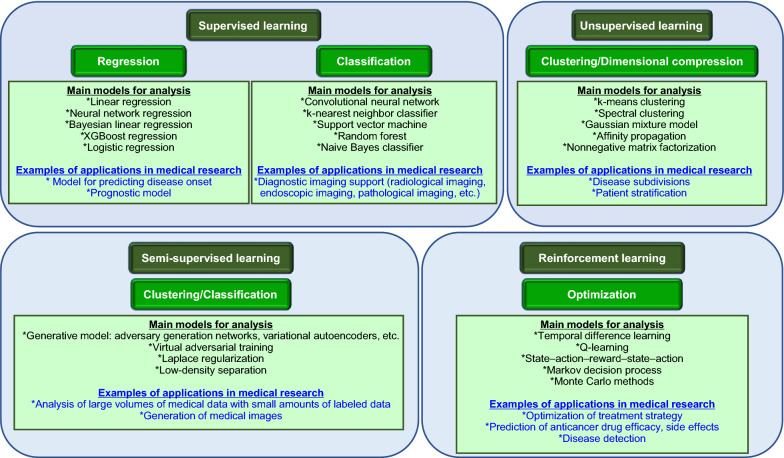
Applications of machine learning technology. The main models used in the analysis of each objective and their application to medical research are shown. The model is also used for various other aspects, for example, support vector machine is used for regression as well as classification

Current AI has become vastly popular due to its basis on machine learning with deep learning as its technological foundation [30, 50]. Based on its analytical characteristics, machine learning can be broadly classified into four categories: supervised, unsupervised, semi-supervised, and reinforcement learning (Fig. 1) [51, 52]. Supervised learning is then broadly classified into regression and classification problems, and regression models are used as models for disease onset prediction and prognostic prediction [53–55]. Classification by supervised learning is currently the most widely studied method, especially in medical image analysis, and is practically applied in clinical practice [33, 56–63]. This is because deep learning technology is particularly strong in image analysis, and when medical AI is practically applied to clinical practice, it is important to use data obtained from physicians’ diagnoses (especially specialists) as training data. Unsupervised learning is subsequently used for clustering and dimensionality compression, and its applications in medical research include patient stratification, medical image categorization, and disease subdivision [64–68]. Semi-supervised learning combines supervised and unsupervised learning. This technology has attracted attention in the medical field because it enables highly accurate learning even with limited amount of training data available. After supervised learning with limited labeled data using this method, it becomes possible to perform analysis based on unsupervised learning with a large amount of unlabeled data and generate medical images using the generated model [69–73]. Lastly, reinforcement learning is a technique that continuously learns from each experience to optimize subsequent behavior and maximize the final outcome. The algorithm of AlphaGo, the AI that defeated the world’s top Go players, uses a learning method called Q-learning, which is a type of reinforcement learning with the optimal action value defined as Q-value. The algorithm selects the action that maximizes the Q-value from a large number of trials and their results [74, 75]. In medical research, it is used for disease detection, optimization of treatment strategies, and prediction of efficacy and side effects of anticancer drugs [76–80].

## Medical applications of computer-aided diagnosis support and NLP

Computer-aided detection/diagnosis (CAD) is important in describing AI-based diagnostic assistance. CADe (computer-aided detection) is a device that incorporates a software that allows a computer to automatically detect and mark the location of candidate lesions on an image, and the computer processes medical images and inspection data if possible to assist in the detection of lesions or abnormal values [81]. CADx (computer-aided diagnosis) is a stand-alone software or device with a software that detects suspected lesion sites, outputs quantitative data as numerical values and graphs, such as discrimination of lesion candidates as good or bad and the degree of disease progression [82], including those that provide diagnostic support by providing candidate diagnostic results, information on risk assessment, etc.

In the late 1950 s, during the dawn of modern computers, studies examined the development of CAD [83–85]. In the 1970 s, there was substantial worldwide interest in expert systems, and the MYCIN expert system was developed as an early CAD system. MYCIN is generally recognized as one of the most important early applications of AI in the medical field [86]. It uses a simple inference engine and has a knowledge base of ~ 500 rules. Several simple “yes/no” or written response questions are asked by the physician, and a final list of possible causative bacterial names (in order of probability), the level of confidence in each, its reason, and the recommended course of drug therapy are determined. Since it showed a relatively good diagnostic accuracy of ~ 65% in clinical cases [87], further refinement of the algorithm was expected to lead to more accurate diagnoses in various areas, leading to many research and development efforts aimed at applying the expert system to medicine. However, MYCIN was never applied clinically because computer performance was poor at that time, and the social infrastructure, such as laws and bioethics, had not been developed. The development of an expert system requires a substantial amount of time and effort; the expert system cannot convert ambiguous human expressions into rules, resulting in inconsistencies, and other fundamental shortcomings [30, 88].

Subsequently, in the late 1980 s and early 1990 s, the focus shifted to the use of data mining approaches for more sophisticated and flexible CAD systems. A significant milestone was achieved in 1998 when the first commercial CAD system for mammography, the ImageChecker M1000 system, was approved by the U.S. Food and Drug Administration (FDA) [89, 90]. Following this, CAD for detecting lung cancer (nodule shadows) in plain chest radiographs and chest CT images, and for polyp detection in colon CT examinations received FDA approval in rapid succession [91–93].

In the 21st century, with the development of deep learning technology using autoencoder by Hinton et al., image analysis using AI technology began to attract attention [94]. This trend has also been observed in the medical field, and the focus is on the development of CAD systems that take advantage of AI. Particularly in 2015, in the ImageNet Large-Scale Visual Recognition Challenge (ILSVRC), an object recognition rate competition, a group from Microsoft demonstrated recognition accuracy with lesser than the average human error rate of 4.9% by using deep learning technology. Furthermore, in 2017, AlphaGo was named the world’s top performer in the game of Go, and there were a number of reports of AI surpassing human capabilities [95–97]. These facts spurred the development of the AI-based software as a medical device (SaMD). More than 300 AI-SaMDs have been approved by the U.S. FDA so far, and clinical applications are being explored, focused on medical image (radiological, endoscopic, ultrasound, etc.) analysis [98, 99]. Additionally, AI is also being introduced for analysis of omics, and medical information and research papers [100–105].

NLP, a branch of artificial intelligence, is a series of techniques that allows computers to process everyday human language [106]. With the recent developments in deep learning technology, it is becoming possible for machines to understand and translate natural language [107]. NLP is also being actively studied in the medical field, as a significant portion of its diverse data generated contains such sentences [108–112]. Until recently, secondary use of medical data had been primarily based on relatively structured data, such as health checkup and medical fee data, but recently it is being developed for handling larger-scale, unstructured data. We categorize the utilization of natural language data in the medical field into three major trends. The first is toward the utilization of data from physicians’ daily practice, represented by medical records (electronic) [113–115]. For example, automatic extraction of adverse drug reaction signals from electronic medical records is being attempted [116–118]. The second major trend is the use of NLP to analyze published data, such as medical articles and case reports, to extract important information for clinical applications [119–121]. In particular, a vast number of medical papers and case reports are published daily, and it is physically impossible for a clinician to comprehensively examine them [110]. Therefore, we believe that the extraction of important information using NLP is rather essential. The third major trend, which has been gaining attention over the past few years or so, is toward the private data that patients exchange through social media and patient associations [122–126]. The NLP system extracts episodes related to patients’ treatment, problems, and practical knowledge obtained from texts via social media. By creating and providing content with appropriate medical information to the extracted episodes, and building a mechanism for sharing similar episodes and practical knowledge among patients, an attempt is being made to create a foundation for patients and medical professionals to learn and utilize this information to enhance patient care.

Therefore, it is important to use NLP appropriately when considering MTB efficiency.

## General MTB tasks and workflow

MTB, a meeting held to medically interpret the clinical implications of the results of genetic analysis obtained using NGS with the aim of proposing appropriate treatments for each individual patient, is critically important in promoting precision medicine [127–132]. Table 1 describes the general workflow of the MTB, showing an example of the MTB conducted at the National Cancer Center (NCC) Japan. Although the databases used and other details vary by country and institution, the basic work performed is common. In addition, Table 2 introduces databases that are important for MTB.

**Table 1:** Example of MTB workflow

	Work item	Work details
I	Assign biological significance to the genetic abnormality (e.g., whether it contributes to the acquisition of a particular trait, such as oncogenic potential).	Focusing on variants whose pathological significance is judged differently in laboratory company reports and C-CAT survey results, the registration statuses of the Gene Polymorphism Database (gnomAD), Somatic Mutation Database (COSMIC), and ClinVar will be checked to determine their pathological significance.
II	Interpretation of genetic evidence for diagnosis and prognosis.	Search public databases (CIViC, OncoKB, etc.) and literature for information on diagnosis and prognosis.
III	Attach a specific candidate drug and level of evidence corresponding to the genetic abnormality after considering basic patient information (cancer type)	Focus on drugs listed in laboratory company reports and C-CAT survey results, and search public databases (CIViC, OncoKB, etc.) and literature to confirm the level of evidence based on the latest findings.
IV	When germline gene abnormalities are recognized (or suspected), the significance and response should be discussed in accordance with relevant guidelines, guidance, and recommendations.	Consider significance and response based on guidelines, guidance, and recommendations related to secondary findings.
V	After considering basic patient information including previous history of chemotherapy, specific candidate drugs and evidence levels corresponding to the genetic abnormality, as well as their approval status and clinical trial status, should be investigated.	Review the list of clinical trials being conducted at the hospital and consider whether to enroll the patient in the relevant clinical trial.
VI	If necessary, consider whether any of the candidate drugs listed in IV can be recommended, considering the patient’s condition, availability, etc.	Check the pathological significance of each gene, level of evidence for the drug linked to the genetic mutation, and availability of the drug.

**Table 2:** Databases commonly used in MTB

Database name	Operating organization [URL]*	Database contents	References
ClinVar	NCBI [https://www.ncbi.nlm.nih.gov/clinvar]	This database collects information on the diversity of the human genome and related diseases and provides it as a freely available archive. It contains polymorphism locations, gene names, and their relationship to diseases. Gene information is linked to dbSNP and dbVar at NCBI, and phenotypes are linked to MedGen. Information is also collected from the NIH Genetic Testing Registry (GTR), OMIM, and PubMed. Each data can be viewed in HTML format or downloaded in XML or tab-delimited format, and some data in VCF files.	[209]
COSMIC	Wellcome Trust Sanger Institute [https://cancer.sanger.ac.uk/cosmic]	COSMIC is the most detailed and comprehensive resource for studying the effects of somatic mutations in human cancer. In addition to coding mutations, COSMIC covers all genetic mechanisms by which somatic mutations promote cancer, including non-coding mutations, gene fusions, copy number mutations, and drug resistance mutations. The core COSMIC database is complemented by additional datasets that allow users to contextualize the biomarkers they detect. The Cancer Gene Census provides a detailed catalog of > 700 genes involved in cancer, their biological functions, and descriptions of the genetic mechanisms that cause cancer. The Cancer Mutation Census provides information on the significance of all coding mutations based on biological and biochemical information from multiple sources. Mutation Actionability in Precision Oncology (Actionability) provides updates on drugs targeting specific somatic mutations at all stages of development. The Cell Lines Project advocates cell line omics data through systematic characterization of the genetics and genomics (variation and gene expression) of over 1,000 cancer cell lines.	[210]
gnomAD	Broad Institute [https://gnomad.broadinstitute.org]	gnomAD is a resource developed by an international coalition of researchers with the goal of aggregating and harmonizing exome and genome data from various large-scale sequencing projects and making them available to a wider audience of scientists. The v2.1.1 dataset (GRCh37/hg19) provided by this site covers 125,748 exome sequences and 15,708 whole genome sequences of unrelated individuals sequenced as part of various disease-specific and population genetic studies (as of March 2022). v3.1.2 dataset (GRCh38) has 76,156 genomes selected (as of March 2022), similar to v2.	[211]
OncoKB	Memorial Sloan Kettering Cancer Center [https://www.oncokb.org]	OncoKB is a groundbreaking precision oncology knowledge base that leverages the clinical expertise of Memorial Sloan Kettering (MSK) to provide accurate and up-to-date information on the biological and clinical significance of > 5,000 cancer gene mutations. On October 7, 2021, the U.S. Food and Drug Administration approved OncoKB for partial listing as the first tumor mutation database to be included in the Public Human Gene Mutation Database. This is the first tumor mutation database to be recognized by the FDA. Treatment information is categorized by the OncoKB Levels of Evidence system, which assigns clinical utility (from standard to investigational treatment) to individual mutational events.	[212]
CIViC	The McDonnell Genome Institute at Washington University School of Medicine [https://civicdb.org/home]	CIViC is an expert knowledge base for the clinical interpretation of variants in cancer, describing the therapeutic, prognostic, diagnostic, and predispositional relevance of all types of genetic and somatic mutations. CIViC is committed to open source code, open access content, public application programming interfaces (APIs), and proof of supporting evidence, enabling the transparent creation of current and accurate variant interpretations for use in precision medicine for cancer.	[213]
ClinicalTrials.gov	U.S. National Library of Medicine [https://www.clinicaltrials.gov]	ClinicalTrials.gov is a registry of clinical trials. Operated by the National Library of Medicine (NLM) of the U.S. National Institutes of Health, it is the largest clinical trials database with > 408,000 registered clinical trials in 220 countries. ClinicalTrials.gov was created as a result of the Food and Drug Administration Modernization Act of 1997 (FDAMA), which required the U.S. Department of Health and Human Services (HHS), through the NIH, to verify the effectiveness of experimental drugs for serious or life-threatening diseases and conditions by using the Clinical Trials. The NIH and the FDA jointly developed the site, which became available to the public in February 2000, to establish a registry of information on federal and private clinical trials conducted pursuant to an application.	[214]
BRCA Exchange	Global Alliance for Genomics and Health [https://brcaexchange.org/]	This database aims to improve our understanding of the genetic basis of breast, ovarian, pancreatic, and other cancers by compiling BRCA1/2 gene mutations and corresponding clinical data from around the world. It is possible to search for BRCA1 or BRCA2 variants online.	[215]
LOVD	Molecular Health [https://www.lovd.nl/]	The database provides a flexible and freely available tool to display gene-centric collections and DNA mutations. It also provides storage for patient-centric and NGS data, and extragenic mutations. LOVD is open source, released under the GPL license, and is being actively improved.	[216]

*This information is current as of September 2022

The first important step in obtaining data from patient-derived samples analyzed using NGS is to assign biological significance to the genetic abnormality (e.g., whether it contributes to the acquisition of a particular trait, such as oncogenic potential). In the NCC Japan, focusing on variants whose pathological significance is judged differently in laboratory company reports and survey results of Center for Cancer Genomics and Advanced Therapeutics (C-CAT) [133], the registration status of gene polymorphism database (gnomAD), somatic mutation database (COSMIC), and ClinVar, a public database of variant interpretations, will be checked to determine the pathological significance of the final judgment.

This is followed by interpretation of genetic evidence for diagnosis and prognosis. Here, public databases (CIViC, OncoKB, etc.) and literature are searched to determine if there are any findings regarding diagnosis and prognosis.

The next step is to attach specific candidate drugs and evidence corresponding to the genetic abnormality, considering basic patient information (cancer type). Here, the level of evidence based on the latest findings was confirmed by searching public databases (CIViC, OncoKB, etc.) and literature, focusing on drugs listed in laboratory company reports and C-CAT survey results. When germline gene abnormalities are present (or suspected), the significance and response should be based on guidelines, guidance, and recommendations related to secondary findings. The list of clinical trials being conducted at the hospital will be reviewed and the possibility of enrollment in the relevant clinical trials will be considered.

If necessary, the specific candidate drugs listed will be reviewed to see if any of them are recommended for the patient’s condition and availability. Here, the pathological significance of each gene, level of evidence for the drug linked to the genetic variant, and availability of the drug are identified.

The OncoKB database is used as an example for further details (as of September 2022) [134] because interpretation of evidence for genetic abnormalities is important [135]. First, FDA-approved biomarkers that predict response to FDA-approved drugs are level 1, with 44 registered genes. Then the FDA-approved standard of care biomarker that predicts response to the drug is at level 2, with 23 registered genes. There is strong clinical evidence showing that the biomarkers are predictive of response to drugs, but neither the biomarkers nor the drugs are standard of care at Level 3, with 33 registered genes. In addition, there is strong biological evidence showing that a biomarker predicts response to drugs, but neither the biomarker nor the drug is a standard treatment, which is level 4, with 25 genes registered. Furthermore, eight genes are registered at level R1, the FDA-approved standard of care biomarker for predicting resistance to drugs.

These are the series of MTB flows; however, with the increasing expectations for precision medicine, increasing burden on MTB members, and aim to conduct more efficient and comprehensive surveys, attempts are being made to introduce state-of-the-art AI and ICT technologies into MTB. The next section introduces research results using AI technology that could be applied to each task in MTB.

## AI-based prediction of biological significance for genetic abnormalities and its application to diagnosis and proposal of candidate drugs for treatment

**Fig. 2:**
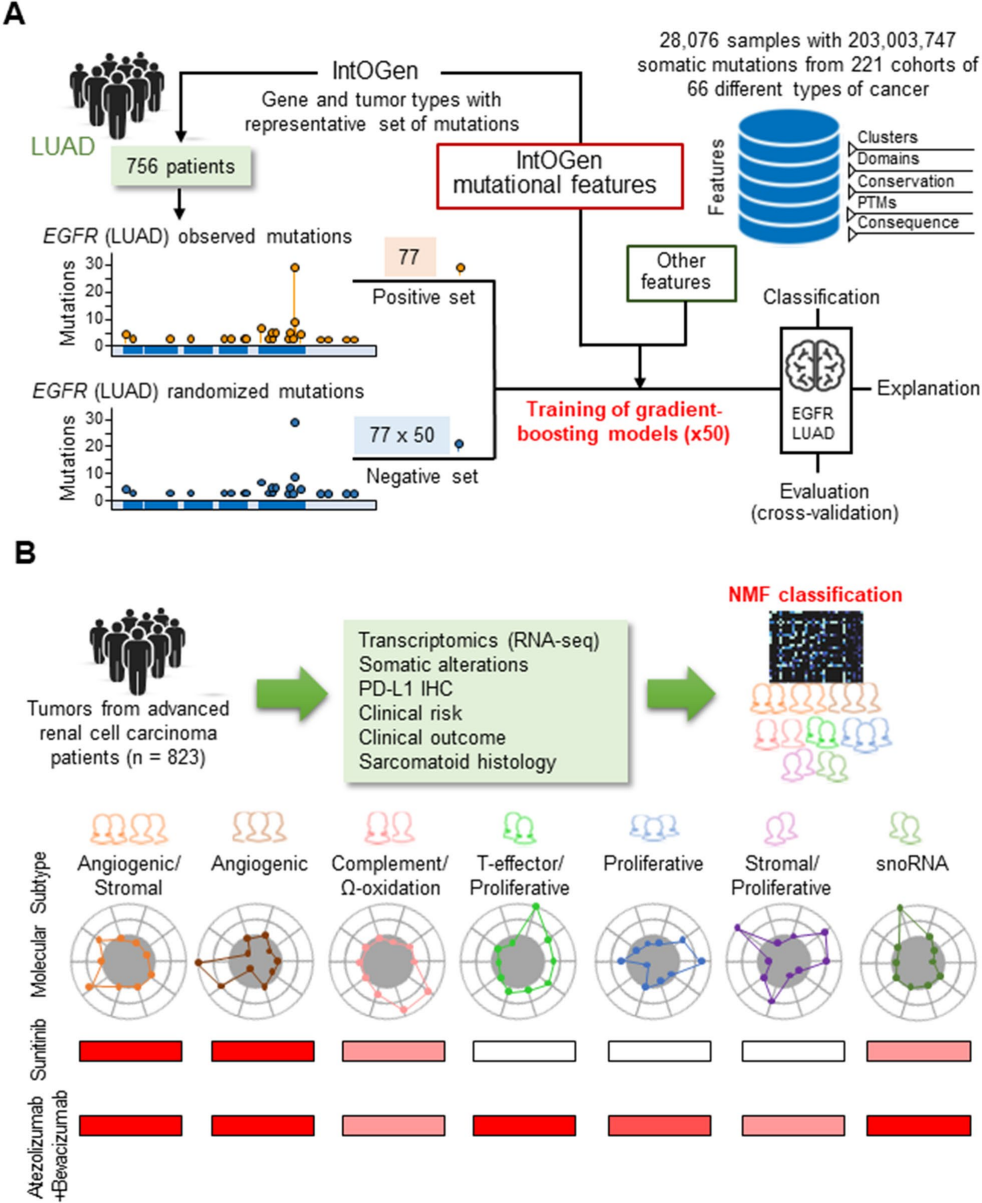
Examples of platforms for the prediction of biological significance for genetic abnormalities and its application to diagnosis. **A** Schematic diagram showing an overview of BoostDM modified from Ref. [136]. A specific model (gradient boosted tree) was constructed for each of the 282 gene-tissue combinations based on 18 features that characterize the mechanism of tumorigenesis of oncogenes. Specifically, 50 basic classifiers were trained on random subsets with equal numbers of positive and negative mutations to adequately represent the diversity of passenger mutations and prevent overfitting. **B **Schematic diagram of the identification of seven molecular subtypes of RCC tumors utilizing machine learning, modified from Ref. [138]. An integrative, multi-omics analysis of 823 tumors from RCC patients identified molecular subsets associated with differences in clinical outcomes with angiogenesis inhibitors alone or in combination with checkpoint inhibitors. Unsupervised transcriptome analysis using NMF revealed seven molecular subsets with different angiogenic, immune, cell cycle, metabolic, and stromal programs

Despite the existence of an excellent database on oncogenes, it is difficult to determine the significance of most of the mutations identified in oncogenes for tumorigenesis, regardless of tumor type. To address this challenge, Muiños et al. developed BoostDM, a machine learning-based methodology for in silico saturation mutagenesis of cancer genes to assess the carcinogenicity of mutations in human tissues (Fig. 2A) [136]. In silico saturation mutagenesis is a term that generally refers to the computational assessment of all possible changes in a gene or protein. BoostDM defines a supervised learning strategy based on mutations observed in sequenced tumors and their annotation by site-specific mutation features, comparing mutations observed in genes with sufficiently high result type-specific excess (by dNdScv) with randomly selected mutations according to three-base mutation probabilities. This method examines the protein-coding sequence of the genome, and all considered mutations are mapped to the canonical transcript of the protein-coding gene according to Ensembl Variant Effect Predictor (VEP.92) [137]. Gene-tumor type-specific BoostDM models can be complemented with other models trained on pooled mutations from relevant tumor types and used to classify mutations observed in a patient’s tumor into drivers and passengers, an important step toward precision cancer medicine. According to the ClinVar and OncoKB databases, only 6,886 and 5,136 (12% and 9%) of the 55,729 coding variants in 568 cancer genes in 28,076 tumor samples are considered drivers (pathogenic or potentially pathogenic) or passengers (benign or potentially benign), respectively. In contrast, more than half of the mutations can be interpreted using the BoostDM model (26% via gene-tumor type-specific models). The BoostDM model is incorporated into the cancer genome interpreter (CGI; https://www.cancergenomeinterpreter.org/), a system designed to assist in the interpretation of newly sequenced tumor genomes.

Motzer et al. performed an integrative, multi-omics analysis of 823 tumors from patients with advanced renal cell carcinoma (RCC) and identified molecular subsets associated with differences in clinical outcomes with angiogenesis inhibitors alone or in combination with immune checkpoint inhibitors (Fig. 2B) [138]. In this study, to better understand the biology of RCC, an RNA-seq dataset of 823 tumor samples from patients with advanced RCC, including 134 tumor samples with sarcoma-like features, obtained from a randomized international phase III trial (IMmotion151) [139], was used to classify patients into seven clusters by utilizing non-negative matrix factorization (NMF). NMF is a machine learning method [140], and unsupervised clustering was used to identify subtypes with different angiogenesis, immunity, cell cycle, metabolism, and stroma programs. Results showed that VEGF receptor tyrosine kinase (sunitinib) and angiogenesis inhibitor (bevacizumab, anti-VEGF) + immune checkpoint inhibitor (atezolizumab) were effective in subsets with high angiogenesis, and bevacizumab + atezolizumab had improved clinical efficacy in tumors with high T effectors and cell cycle transcription. Somatic mutations in the PBRM1 and KDM5C genes were associated with high angiogenesis and AMPK/fatty acid participation gene expression, while changes in CDKN2A/B and TP53 were also associated with an increased cell cycle and anabolic metabolism. Sarcomas have a lower prevalence of PBRM1 mutations and angiogenic markers, higher frequency of CDKN2A/B mutations, and increased PD-L1 expression. These findings can be applied to the molecular stratification of patients, improving the prognosis of sarcomas by combining checkpoint inhibitors with angiogenesis inhibitors, and developing personalized medicine in RCC and other indications.

**Fig. 3:**
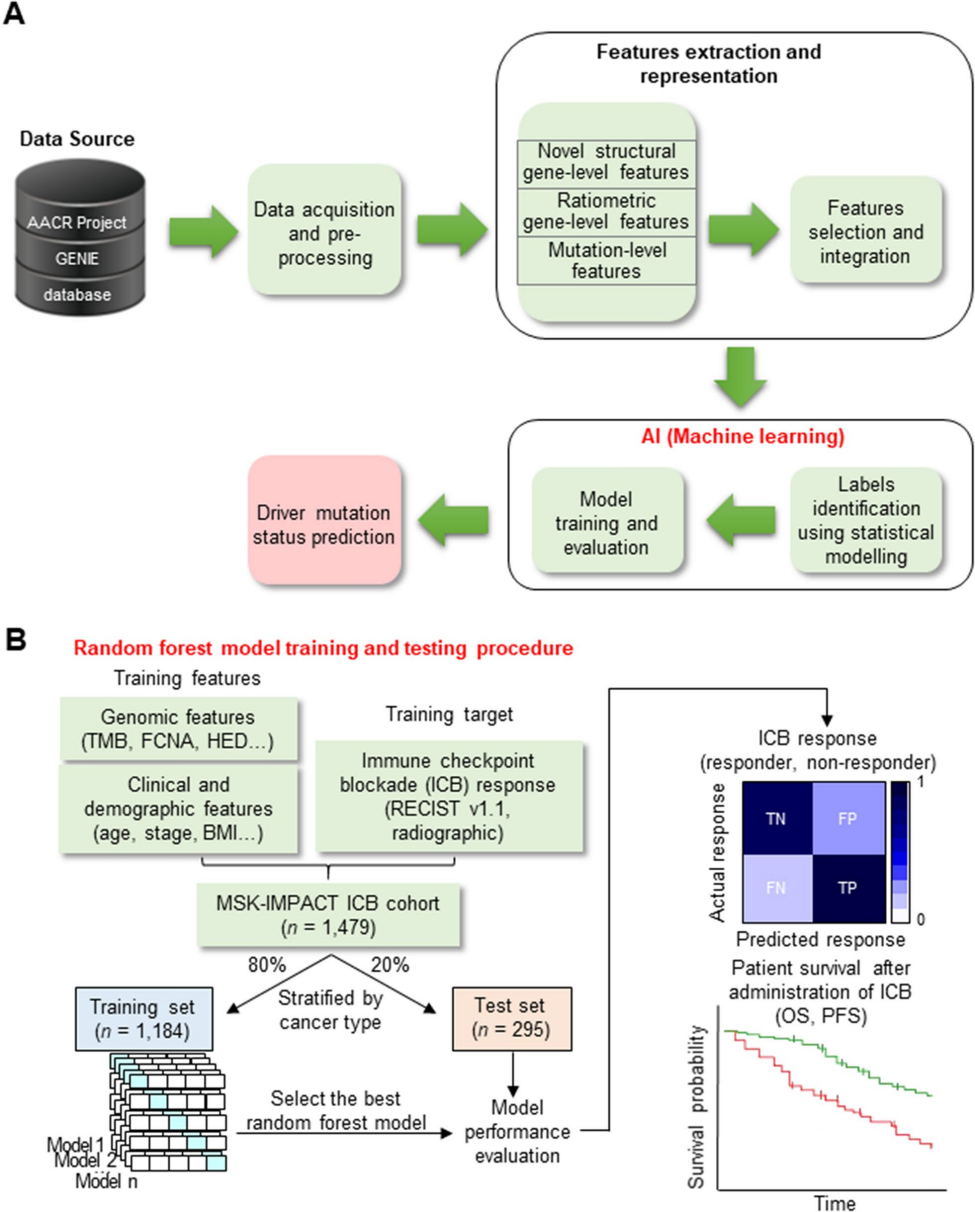
Introduction of a machine learning-based genetic mutation analysis platform. **A** Schematic diagram showing an overview of DRIVE, a feature-based machine learning platform for pan-cancer assessment of somatic missense mutations, modified from Ref. [142]. This approach uses a total of 51 features spanning the gene and mutation levels. Several state-of-the-art supervised machine learning algorithms were applied to the final dataset, with results presented for the highest performing algorithms, including random forests, logistic regression, extreme gradient boosting, k-nearest neighbors, support vector machines, and multilayer perceptron. **B** Figure outlining a machine learning model for predicting response to immune checkpoint inhibitors, modified from Ref. [147]. Sixteen cancers were individually divided into training (80%) and testing (20%) subsets. To predict (responder and non-responder) immune checkpoint inhibitors, random forest models were trained on multiple genomic, molecular, demographic, and clinical characteristics on the training data using fivefold cross-validation. Consequently, trained models with optimal hyperparameters were evaluated on various performance measures using the test set

Substitutional mutations in tumors have been reported to account for 95% of somatic mutations, 90% of which are missense mutations [141]. Substitutional mutations are further classified into driver mutations that favor cancer cell growth and passenger mutations that do not contribute to growth. Since the emergence of driver mutations and cancer heterogeneity are key factors in overcoming treatment resistance and treatment failure, distinguishing whether a substitution mutation is a driver or passenger mutation is an important challenge. Therefore, Dragomir et al. developed and reported a new method (DRIVE) that utilizes machine learning techniques to identify driver and passenger mutations (Fig. 3 A) [142]. Mutation-level characteristics are based on pathogenicity scores, while gene-level characteristics include the maximum number of protein-protein interactions, biological processes, and types of post-translational modifications. To validate the ability of the proposed method, it was evaluated on a benchmark dataset, which showed that both gene- and mutation-level features were representative of driver mutations, and the proposed method was > 80% accurate in finding the true mutation type. The results suggest that machine learning methods can be used to gain knowledge from mutation data to achieve more targeted cancer treatments [142].

Cancer immunotherapy, represented by immune checkpoint inhibitors, is a treatment that can induce the immune system to effectively recognize and attack tumors. The main approved drugs are antibodies that target CTLA-4 and PD-1/PD-L1 and can induce sustained responses in patients with advanced cancer [143, 144]. However, clinical benefit has not been achieved in many patients, highlighting the need to identify patients who will respond to immunotherapy [145, 146]. Chowell et al. integrated genomic, molecular, demographic, and clinical data from a comprehensive curated cohort (MSK-IMPACT) of 1,479 patients treated with immune checkpoint inhibitors in 16 different cancer types to develop a machine learning platform (Fig. 3B) [147]. Using random forests as the machine learning technique, the platform achieved high sensitivity and specificity in predicting clinical response to immunotherapy in a retrospective analysis, predicting both overall survival (OS) and progression-free survival in test data across different cancer types. The analysis platform also significantly outperforms the tumor mutation burden-based predictions recently approved by the U.S. FDA for predicting immune checkpoint inhibitor responses and can quantitatively assess the most salient model features for prediction.

## Integrated analysis of EHR data using AI and its application to diagnosis and treatment

**Fig. 4:**
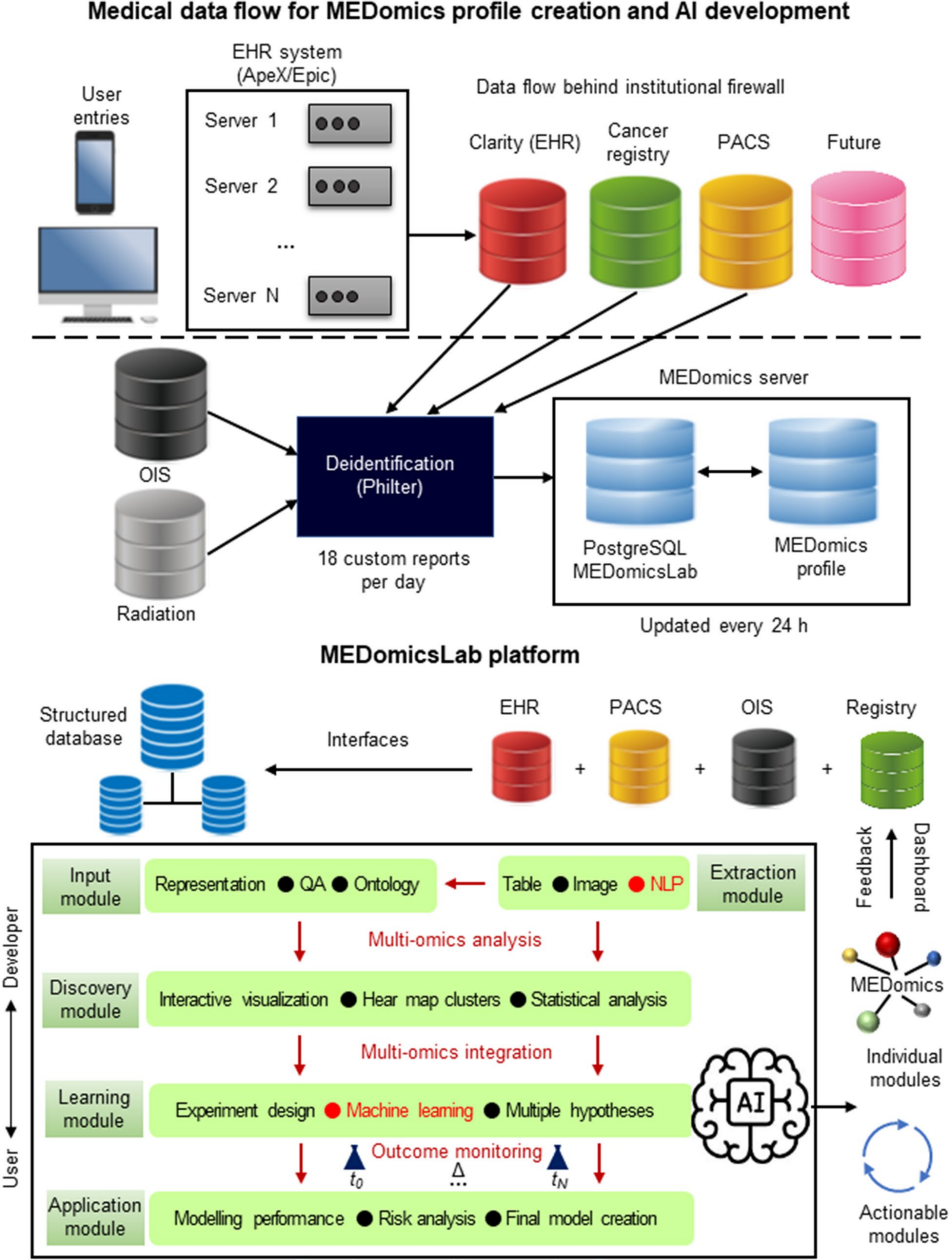
Schematic diagram of the flow of medical data for the creation of MEDomics profiles and AI development, modified from Ref. [150]. Medical information generated by medical activities is continuously recorded in the Clarity (EHR) relational database. A custom report is generated from Clarity and sent to the MEDomics server for personal identification, data formatting, and feature extraction and calculation. Various other databases are processed by MEDomics, including tumor information systems (radiation), treatment planning systems, and imaging. Data are ultimately recorded and updated daily on a MEDomics server located behind the facility’s firewall with dual authentication access. Medical information flowing from hospital databases is collected in a structured database and becomes input to the MEDomicsLab platform. This information is integrated and processed by the MEDomicsLab engine and used in five computational modules: input, extraction, discovery, learning, and application. This creates a statistical model for Precision Oncology that can be returned to the hospital’s database to aid in clinical decision making

Modern healthcare systems generate and store vast amounts of digital information and have great potential for personalizing and improving healthcare delivery [148, 149]. Morin et al. developed a secure, comprehensive, dynamic, and scalable infrastructure called MEDomics designed to continuously capture multimodal electronic medical information across large, complex healthcare networks (Fig. 4) [150]. MEDomics maintains structural data that encapsulates the entire timeline of a particular individual’s medical care, and this cross-sectional profile can be used to develop a variety of AI applications aimed at practical interventions that can be returned to the healthcare system. Utilizing the MEDomics profile, an institution-wide mortality study in breast and lung cancer patients revealed correlations of mortality by stage and other factors consistent with the published literature. The impact of targeted and immunologic therapies on survival in metastatic breast and lung cancer patients was also investigated. In addition, this infrastructure allowed us to investigate the impact of previously reported non-oncologic risk factors, such as the Framingham cardiovascular risk score, on mortality in cancer patients. This indicates that MEDomics is not only useful for continuous learning, but also for generating and testing clinical hypotheses. Importantly, the study also used statistical learning to create a prognostic model to predict mortality with a high degree of accuracy. Furthermore, utilizing a chronological natural language processing approach, more electronic medical records were incorporated as the course of an individual’s illness progressed, and accuracy was found to improve over time. Based on these results, we believe that an approach that combines structured and unstructured multimodal health information in a longitudinal context has the potential to facilitate the development of predictive and dynamic AI applications in oncology that improve the quality and duration of life for individuals.

**Fig. 5:**
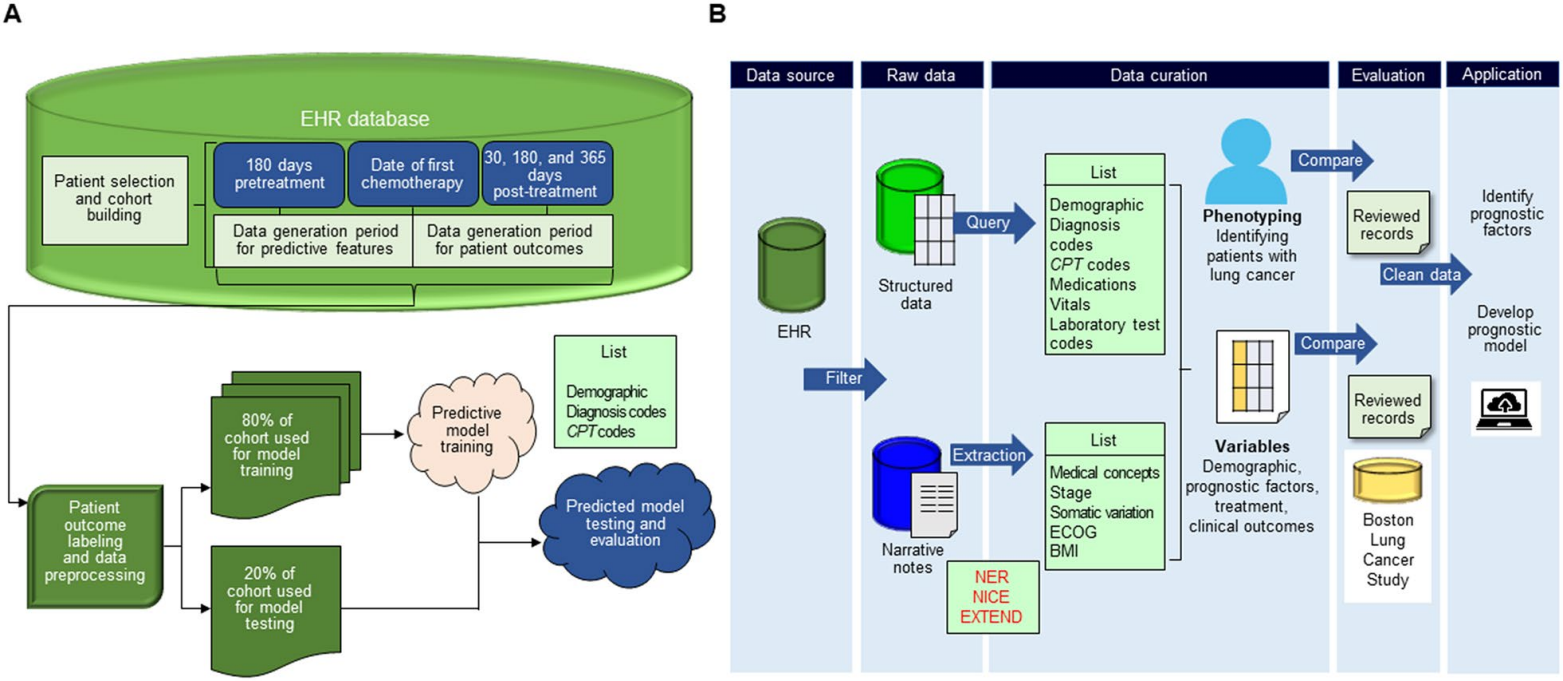
Integrated analysis of EHR data using AI and introduction of its application to diagnosis and treatment. **A** Figure showing a series of processes to predict the risk of preventable ACU after chemotherapy initiation using the machine learning algorithm trained on comprehensive EHR data, modified from Ref. [151]. Nine machine learning models were developed, validated, and compared to predict ACU at 3, 6, and 12 months after chemotherapy initiation in patients presenting to an oncology clinic affiliated with a large academic cancer center. Patient-reported outcomes were also incorporated to assess the impact of these data in predicting the risk of preventable ACU. **B** Schematic of a lung cancer prognostic study using a clinical cohort constructed from EHR data, modified from Ref. [158]. Initially, data are obtained from the EHR and lung cancer diagnosis codes are used as filters. After creating a data mart containing structured data and narrative notes, the structured data are queried and the narrative notes are processed using NLP tools. A phenotyping algorithm has been developed using a combination of structured data and narrative notes to extract variables of interest. The performance of the phenotyping algorithm is compared to a random sample of patients selected for EHR review. The performance of the phenotyping algorithm is compared to a random sample of patients selected for EHR review. The accuracy of the extracted variables is compared to the EHR reviewed sample and the Boston Lung Cancer Study cohort data

Peterson et al. proposed a model to predict the risk of preventable acute care unit (ACU) after chemotherapy initiation using a machine learning algorithm trained on comprehensive electronic health record (EHR) data (Fig. 5 A) [151]. ACU, including emergency department visits and hospitalizations, accounts for approximately half of all cancer care-related costs in the United States [152, 153]. Not only is ACU costly, but unscheduled ACU negatively impacts a patient’s quality of life and result in poor quality care [154, 155]. To improve quality of care, increase transparency, and reduce costs, the Centers for Medicare & Medicaid Services (CMS) introduced the chemotherapy measure (OP-35) [156, 157]. Peterson et al. successfully identified patients at high risk for preventable acute care, the target of the CMS’ OP-35 measure, using machine learning models trained on routinely collected medical information, which showed strong predictive performance. After obtaining structured EHR data generated prior to chemotherapy treatment, 80% of the data in the cohort was used to train a machine learning model to predict the risk of ACU after chemotherapy initiation. The remaining 20% of data were used to test the performance of the model by area under the receiver operating characteristics (AUROC) curve. The study included 8,439 patients, 35% of whom developed preventable ACU within 180 days of starting chemotherapy. In the proposed model, patients at risk of preventable ACU were classified by an AUROC of 0.783 (95% CI, 0.761–0.806) [151]. Patients who were hospitalized were identified better than those who visited the emergency room, and key variables included previous hospitalizations, cancer stage, race, laboratory values, and a diagnosis of depression. The analysis showed limited benefit from the inclusion of patient-reported outcome data and demonstrated inequities in outcome and risk models for Black and Medicaid patients. These results indicate that detailed EHR data can be used to identify patients at risk of ACU using machine learning, and the model proposed in this study has the potential to improve cancer treatment outcomes, patient experience, and costs by enabling targeted preventive interventions [151].

In a cohort study of 42,069 lung cancer patients, Yuan et al. extracted key cancer characteristics from structured data and narrative notes by developing a customized NLP tool using EHRs (Fig. 5B) [158]. Predictive analytics research solution and execution (PheCAP) [159] version 1.2.1 was used as the phenotyping program in this study to develop and evaluate an algorithm to classify lung cancer status. PheCAP consists of three main steps: feature extraction based on the Surrogate-Assisted Feature Extraction (SAFE) algorithm, algorithm development based on penalized regression, and algorithm validation to evaluate the accuracy of the algorithm. The initial PheCAP feature data also consisted of coded features identified by domain experts, NLP features identified from online knowledge source articles proposed in SAFE, and medical utilization features measured by total counts of medical notes. After extracting eastern cooperative oncology group (ECOG) performance status and body mass index information using an electronic medical record numerical data extraction tool, the NLP interpreter for cancer extraction (NICE) tool was developed to infer cancer characteristics, such as stage, histology, diagnosis date, and somatic mutation information, from clinical records including pathology reports, discharge summaries, and progress notes (Fig. 5B). Smoking status is predicted using a classification algorithm. Importantly, the prognostic ability of the final model proposed in this study was statistically significantly superior to the base model AUROC, including gender, age, histology, and stage (1-year prediction: 0.774 [95% CI, 0.758–0.789]; *P* < 0.001; 2-year prediction: 0.779 [95% CI, 0.765–0.793]; *P* = 0.002; 3-year prediction: 0.780 [95% CI, 0.766–0.796]; *P* = 0.002; 4-year prediction: 0.782 [95% CI, 0.767–0.797]; *P* = 0.001; 5-year prediction: 0.782 [95% CI, 0.768–0.798]; *P* < 0.001). In the test set, the final and basic models had C-indexes of 0.726 and 0.697, respectively. On the calibration plots, the measured probability of OS was generally within 95% CI of the predicted probability of OS. EHRs provide a low-cost means of accessing detailed longitudinal clinical data from large populations, and lung cancer cohorts constructed from EHR data have shown the potential to be a powerful platform for clinical outcomes research.

## AI-based medical article retrieval

In the process of an MTB, it is necessary to refer to the literature when interpreting genetic evidence regarding diagnosis and prognosis, considering basic patient information (age, gender, cancer type, etc.) to address genetic abnormalities and attaching specific candidate drugs and evidence levels [160–162]. On the other hand, the number of medical papers published to date is substantial, and it is a difficult task for humans to extract important information from them. Therefore, research has been conducted to efficiently extract useful information from medical papers using NLP [163–165], one of the AI techniques, and we introduce some recent representative results.

**Fig. 6:**
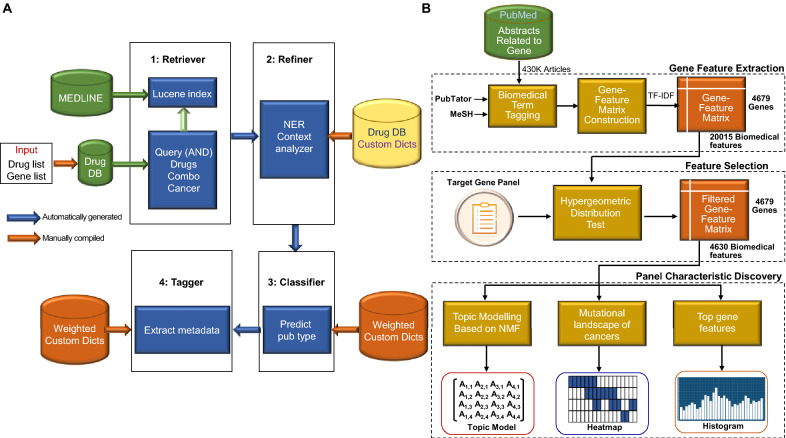
Introduction of AI-based technology for extracting information from medical articles. **A** Schematic diagram modified from Ref. [166]. RetriLite was customized for combination therapy, and a pipeline consisting of four modules was developed. **B** Schematic diagram modified from Ref. [168]. A gene panel analysis framework was developed that can discover gene panel characteristics based on BioNLP.

Zeng et al. developed RetriLite, an information retrieval and extraction framework that leverages NLP and domain-specific knowledge bases to computationally identify relevant articles and extract important information [166]. RetriLite features systematically developed automatic query expansion, utilizing domain-specific dictionaries. The National Center for Biotechnology Information (NCBI) Entrez gene database uses gene symbols and aliases, the NCI Thesaurus uses drug names and aliases, and the glossaries developed by major cancer centers use cancer disease dictionaries created by their own institution. It also uses Lucene [167], a state-of-the-art information retrieval library, as the backbone of the application, rendering basic relevance-based ranking, and uses a term frequency, inverse document frequency weighting scheme as the default ranking, where terms matching search terms contribute to a document’s relevance score. In addition, RetriLite has a keyword highlighting feature, which conveniently conveys the hidden knowledge used in creating the extended query and may aid in knowledge discovery. A general named entity recognition mechanism has been developed that uses a dictionary for input, recognizes the relevant entities in the text, and normalizes them using canonical terms. Regarding contextual analysis, text segmentation was applied, and articles where the matched keywords did not appear in the same context were eliminated. Importantly, Zeng et al. customized RetriLite for combination therapy and developed a pipeline consisting of four modules (Fig. 6A) [166]. The “Retriever” is a module that uses gene and drug lists as inputs. For the gene list, it cross-references the institutional drug database to identify clinically available drugs that directly target the gene, along with their aliases. For the drug list, all names are searched, including the aliases associated with each drug. Next, a conjunctive Boolean search query is created, in which three elements coexist: the target drug, concept of cancer, and concept of combination therapy. Keywords related to combination therapy were created by two domain experts. In the second “Refiner” module, the article is considered qualified if it contains at least one sentence in which two drug entries co-occur with the concept of combination therapy, using named entity recognition and contextual analysis to refine the search function. In the third “Classifier” module, a customized weighted terminology dictionary created by the institution is used to classify the main themes of the article as either clinical or preclinical. The fourth “Tagger” module generates relevant metadata tags to facilitate expert review and help navigate the large corpus. For example, tags have been created for general categories of cancer types related to solid tumors and/or hematological malignancies, drugs matching the search query (anchor drugs), other drugs not included in the original search query but recognized in the context of the combination, types of studies (clinical and/or preclinical), and specific safety-related concepts, such as side effects described in the abstract. Regarding the results, RetriLite achieved an F1 score of 0.93 after more extensive validation experiments to identify drugs with enhanced antitumor effects in vitro or in vivo using poly(ADP-ribose) polymerase inhibitors [166]. Of the articles determined to be relevant by this framework, 95.5% are true positives, achieving an accuracy rate of 97.6% with respect to distinguishing between clinical and preclinical articles. It is also worth mentioning that the inter-observer evaluation achieved a 100% agreement rate [166]. These results indicate that RetriLite is an applicable framework for building domain-specific information retrieval and extraction systems, and its extensive and high-quality metadata tags and keyword highlighting may allow more effective and efficient access to combination therapy information.

Chen et al. applied Biomedical Natural Language Processing (BioNLP) techniques to literature mining of cancer gene panels aimed at creating a pipeline that can contextualize genes using text-mined co-occurrence features (Fig. 6B) [168]. The gene panel analysis framework was developed in this study. First, PubMed abstracts that mention genes relevant to humans were extracted. This step filters ~ 430,000 PubMed abstracts on genes from the current full PubMed corpus, which contains ~ 30 million articles. Second, biomedical named entity recognition is performed on the extracted PubMed abstracts using PubTator and Medical Subject Headings. Third, a genetic term-feature matrix was constructed using biomedical terms, with concepts similar to the document-term matrix. Fourth, to ensure the term features generated in the previous step correspond more strongly to the target gene panel, term feature selection is performed for each individual gene panel. An important aspect of this study is the exploration of hypergeometric distribution. By comparing the frequency distribution of each term feature in the target gene set and the total gene set, it is expected that term features that are more correlated with the target gene panel will be enriched. This approach allows for flexibility with respect to different target gene sets, such as the Oncomine Cancer Research Panel (OCP) [169] and cardiovascular gene panels [170]. For results using this framework, the cosine similarity of gene frequencies between text mining and statistical results from clinical sequencing data was 80.8%. In the different machine learning models, the peak accuracies for the prediction of MSK-IMPACT and OCP were 0.959 and 0.989, respectively. Receiver operating characteristic curve analysis also confirmed that the neural network model had better predictive performance (AUROC = 0.992) [168]. By using text-mined post-occurrence features, the literature for each gene can be ascertained, and this approach could be used to evaluate several existing gene panels and predict the remaining genes using a portion of the gene panel set, leading to cancer detection.

## Attempts to support MTB by using AIs

Recent successes of AIs in various fields motivated researchers to develop AIs that support MTBs. It is well known that IBM won the game of Jeopardy! in 2011 with a strong NLP technology. The technology enabled the development of remarkable services called Watson for Oncology and Watson for Genomics, where the former is based on disease history and the later on genomic sequencing. They achieved excellent overall consistencies with human experts while reducing doctors’ efforts [171–173]. In spite of these achievements, they faced difficulties in the messy reality of healthcare system and its performance depends on race, age, and cancer type [174]. Such difficulties might be universal obstacles for any AIs that aim to support MTBs. Another attempt to support MTBs is a cloud-based virtual molecular tumor board (VMTB) that includes a knowledge base, scoring model, rules engine with > 51,000 rules, an asynchronous virtual chat room and a reporting tool [160]. VMTB also reduced time from data receipt to report delivery. In addition, biomarker-driven clinical-trial opportunities were identified for more patients from personalized treatment plans by VMTB than from a commercial lab test alone. However, variability in duration of response to targeted therapy was observed, which might be mitigated with more-explicit consideration of the extent of intra-patient tumor heterogeneity and evolution [175].

## Current challenges and possible future AI-based MTBs

In this review, we introduced the potential of AI implementation in MTBs with a particular focus on the following three areas: (1) AI-based prediction of biological significance for genetic abnormalities and its application to diagnosis and the proposal of therapeutic candidates; (2) AI-based integrated analysis of EHR and omics data and its application to diagnosis and treatment; and (3) AI-based medical article retrieval. Considering the current situation, the use of AI technologies, including machine learning and NLP, is essential for MTBs to proceed smoothly and efficiently, and the active introduction of AI is desirable in the future. On the other hand, there are several issues that must be resolved in the future. The challenges to be addressed are discussed here.

An issue with current cancer genome medicine is the number of patients who can be offered appropriate treatments as a result of genetic testing is limited [176–178]. This is mainly because current cancer genomic medicine is based primarily on a targeted-gene panels coupled with next-generation sequencing (only a limited number of major driver genes are tested). In the future, it is necessary to build a platform for precision medicine based on more omics information, such as whole genome analysis and epigenome information [50, 179]. These are still at the basic research level, and future research aimed at clinical application is desirable. In particular, because a strength of machine learning is its ability to perform multimodal analysis, it is also important to establish a method to integrate and analyze multiple omics information [50].

Second, to date, medical image analysis has been the leading medical AI research and development method, and most AI-based medical device programs approved by the FDA are also targeted at medical image analysis [30, 31, 99, 180, 181]. Compared to medical image analysis, the introduction of AI into omics analysis has not progressed. This is because the nature of omics data itself is difficult to handle, judging from the characteristics of machine learning technology. For example, samples in the medical field are difficult to obtain, and there are limitations on the number of cases that can be analyzed. On the other hand, there are ~ 30,000 genes, which are the central target of omics analysis, and the target of whole genome analysis consists of three billion base pairs. The number of parameters (p) is overwhelmingly large compared to the number of cases, which makes machine learning difficult (called the small n, large p problem) [182–184]. In addition, since neighboring pixels tend to have similar information with respect to images, models such as convolutional neural networks, one of the deep learning techniques, are useful [185, 186]. On the other hand, with respect to genomic information, there is often a divergence between chromosomal location (proximity) and functional relatedness (close proximity between genes does not mean that they are functionally related). Consequently, the usefulness of machine learning in medical image analysis is often not observed in omics analysis. New models and analysis platforms for these problems are being developed by our research group and others [100, 102, 187–190], and it is hoped that robust systems that can be applied clinically will be developed in the future.

**Fig. 7:**
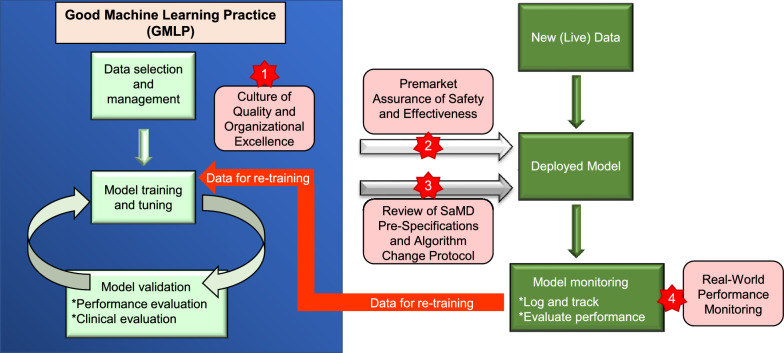
Overlay of FDA’s TPLC approach based on GMLP and artificial intelligence/machine learning modified workflow [196]. This TPLC approach can enable the organization to continuously excel by evaluating and monitoring software products from development to post-sale performance

Third, regarding cancer genome medicine, the volume of data is increasing daily, and the types of anticancer drugs are also increasing; therefore, it would be ideal for AI to continuously learn. On the other hand, the approved AI-SaMDs are basically locked AIs that were approved once they stopped learning, therefore they are not adaptive AIs that can continuously learn [191–193]. Various efforts are currently underway worldwide to address this issue. In 2019 in the United States, the FDA published a discussion paper on the regulation and framework for AI-SaMDs, where it proposed “SaMD Pre-Specifications (SPS)”, which describe the expected or planned changes to the device, and “Algorithm Change Protocol (ACP)”, a specific proposal on the methods companies should use to manage the risk of change [194]. A concept for the quality control of programmed medical devices called “Good Machine Learning Practice” (GMLP) was also proposed [195]. To limit degradation while allowing machine learning algorithms to completely leverage their power and continuously improve their performance, the total product lifecycle (TPLC) approach proposed by the FDA and based on GMLP is expected to balance benefits and risks, and enable safe and effective AI-SaMD delivery (Fig. 7) [196]. Subsequently, in January 2021, the FDA issued an action paper on AI-SaMD regulations and frameworks [197, 198] and proposed to issue draft guidance on prescribed change management plans. In addition, in October 2021, with the FDA, Health Canada, and the UK’s Medicines and Healthcare products Regulatory Agency jointly identified 10 guiding principles that can inform the development of GMLP and issued a new guidance called “Good Machine Learning Practice for Medical Device Development: Guiding Principles” [199, 200]. It suggests that regular or ongoing training of the model should be managed to prevent overfitting and unintended bias, as well as to place appropriate controls to manage risk. Since adaptive AI is important for the introduction of AI into MTB, progress in this research area is desirable.

Fourth, evidence analysis for genetic abnormalities based on NGS has been reported to vary considerably among annotation services [135, 201, 202]. For example, there is only moderate agreement between IBM Watson for Genomics (WfG) and OncoKB over their Level 1 treatment action likelihood recommendations [135]. This implies that the accuracy of annotation in tumor profiling tests for solid tumors based on genetic mutation analysis by NGS requires improvement.

In addition to the above, other sensitive issues have been reported, such as the fact that the MTB proposal may be out of sync with the actual trial in which the patient can participate because it does not take into account the patient’s medical history (especially drug-induced pneumonitis). Therefore, it is also important that the system can be easily modified to suit the current situation in the clinical field. Furthermore, since this review focuses on the use of AI in MTB, it should also be noted that it presents only limited results among the MTB-related reports so far. Several studies have reported on the various elemental technologies required for MTB, though not specifically focused on AI [203–206], and reviewed AI efforts in clinical diagnosis of cancer (including our previous study) [30, 33, 207, 208].

## Conclusion

This review has shown that AI may be used for various elemental technologies required for MTBs. In particular, the volume of data handled by MTB members is expected to increase in the future, and the introduction of AI into MTB is an urgent requirement to establish a precision medicine system. However, there are several potential challenges of AI, and it is important to progress steadily while solving these challenges individually and simultaneously creating innovative technologies. Imperatively, a win–win relationship between human and AI must be established to create a symbiotic relationship, with a clear understanding of which AI can be beneficial and where it may limit progress.

## Data Availability

Not applicable.

## Abbreviations

MTB: Molecular tumor board
AI: Artificial intelligence
NGS: Next-generation sequencing
ICT: Information and communication technology
C-CAT: Center for Cancer Genomics and Advanced Therapeutics
CAD: Computer-aided detection/diagnosis
CADe: Computer-aided detection
CADx: Computer-aided diagnosis
ILSVRC: ImageNet Large-Scale Visual Recognition Challenge
FDA: Food and Drug Administration
SaMD: Software as a medical device
NCC: National Cancer Center
RCC: Renal cell carcinoma
NMF: Non-negative matrix factorization
OS: Overall survival
ACU: Acute care unit
EHR: Electronic health record
CMS: Centers for medicare & medicaid services
AUROC: Area under the receiver operating characteristics
NLP: Natural language processing
SAFE: Surrogate-assisted feature extraction
ECOG: Eastern cooperative oncology group
NICE: NLP interpreter for cancer extraction
NCBI: National Center for Biotechnology Information
BioNLP: Biomedical natural language processing
OCP: Oncomine cancer research panel
VMTB: Virtual molecular tumor board
SPS: SaMD pre-specifications
ACP: Algorithm change protocol
GMLP: Good Machine Learning Practice
TPLC: Total product lifecycle
gnomAD: Genome Aggregation Database
COSMIC: the Catalogue Of Somatic Mutations In Cancer
CIViC: Clinical Interpretation of Variants in Cancer
WfG: IBM Watson for Genomics
NLM: National Library of Medicine
FDAMA: Food and Drug Administration Modernization Act of 1997
HHS: Department of Health and Human Services
